# INTEROCC case–control study: lack of association between glioma tumors and occupational exposure to selected combustion products, dusts and other chemical agents

**DOI:** 10.1186/1471-2458-13-340

**Published:** 2013-04-12

**Authors:** Aude Lacourt, Elisabeth Cardis, Javier Pintos, Lesley Richardson, Laurel Kincl, Geza Benke, Sarah Fleming, Martine Hours, Daniel Krewski, Dave McLean, Marie-Elise Parent, Siegal Sadetzki, Klaus Schlaefer, Brigitte Schlehofer, Jerome Lavoue, Martie van Tongeren, Jack Siemiatycki

**Affiliations:** 1 University of Montreal Hospital Research Centre (CRCHUM), Montreal, Canada; 2 Center for Research on Environmental Epidemiology (CREAL), Barcelona, Spain; 3 Oregon State University, Corvallis, USA; 4 Monash University, Melbourne, Australia; 5 University of Leeds, Leeds, UK; 6 INRETS, Lyon, France; 7 University of Ottawa, Ottawa, Canada; 8 Massey University, Wellington, New Zealand; 9 INRS-Institut Armand-Frappier|, Montreal, Canada; 10 Gertner Institute, Chaim Sheba Medical Center & Tel Aviv University, Tel Aviv, Israel; 11 DFKZ, Heidelberg, Germany; 12 Centre for Human Exposure Science, Institute of Occupational Medicine, Edinburgh, UK

**Keywords:** Brain cancer, Case–control study, International, Job exposure matrix, Risk factor

## Abstract

**Background:**

The aim was to investigate possible associations between glioma (an aggressive type of brain cancer) and occupational exposure to selected agents: combustion products (diesel and gasoline exhaust emissions, benzo(a)pyrene), dusts (animal dust, asbestos, crystalline silica, wood dust) and some other chemical agents (formaldehyde, oil mist, sulphur dioxide).

**Methods:**

The INTEROCC study included cases diagnosed with glioma during 2000–2004 in sub-regions of seven countries. Population controls, selected from various sampling frames in different centers, were frequency or individually matched to cases by sex, age and center. Face-to-face interviews with the subject or a proxy respondent were conducted by trained interviewers. Detailed information was collected on socio-economic and lifestyle characteristics, medical history and work history. Occupational exposure to the 10 selected agents was assessed by a job exposure matrix (JEM) which provides estimates of the probability and level of exposure for different occupations. Using a 25% probability of exposure in a given occupation in the JEM as the threshold for considering a worker exposed, the lifetime prevalence of exposure varied from about 1% to about 15% for the different agents. Associations between glioma and each of the 10 agents were estimated by conditional logistic regression, and using three separate exposure indices: i) ever *vs.* never; ii) lifetime cumulative exposure; iii) total duration of exposure.

**Results:**

The study sample consisted of 1,800 glioma cases and 5,160 controls. Most odds ratio estimates were close to the null value. None of the ten agents displayed a significantly increased odds ratio nor any indication of dose–response relationships with cumulative exposure or with duration of exposure.

**Conclusion:**

Thus, there was no evidence that these exposures influence risk of glioma.

## Background

Brain tumors constitute a mixed group of intracranial neoplasms which could be benign or malignant. Gliomas account for almost 80% of primary malignant brain tumours [1]. Of the six major histological subtypes of glioma, glioblastoma constitutes the highest grade and the most common type, and is associated with very poor survival [1,2]. The incidence of glioma differs according to age, gender, race, ethnicity and country; in many regions slight increases have been reported between the 1970s and 2000, though it is unclear whether this ostensible increase simply reflected diagnostic improvement [3].

Except for ionizing radiation, which is a well-established risk factor for glioma [4,5], the aetiology of this tumor is largely unknown [4,6,7]. Some case–control studies have found a lower risk of glioma among subjects reporting allergies or other atopic conditions [4]. There is inconsistent and largely negative evidence regarding possible associations between smoking, diet or alcohol and gliomas [3,4]. Concerns have been raised about possible effects of exposure to non-ionizing radiation including radiofrequency (RF) fields due to mobile phones or electromagnetic fields (EMF) in the extremely low frequency range (ELF), but the evidence remains inconsistent and uncertain [8-12].

A few studies have reported on glioma risks in relation to occupations or industries, but there has not been any strong consistent pattern across studies. Slight increased risks have been reported among workers in synthetic rubber manufacturing [13], petrochemical refineries [14] and pulp and paper industries [15], as well as among physicians [14,16,17], firefighters [14,17], farmers [16,18] and legal and social services workers [14]. More studies have reported on occupational risks for all brain cancers combined [18-20]. There have been reports of increased risk of brain tumors for polyvinyl chloride production workers [21,22], anatomists, pathologists and embalmers [23-25], painters, machinists, industrial mechanics and plumbers [26,27]. Few studies have delved into the possible associations between specific occupational exposures in those occupations/industries and the risk of brain cancer. There is a need for studies that go beyond the job or industry title to investigate brain cancer risks in relation to chemical exposures.

The INTEROCC study was built on two important databases: the INTERPHONE study, the largest international collaborative case–control study of brain cancer yet conducted [28], and FINJEM, a database permitting the translation of job histories into occupational exposure histories [29]. The INTERPHONE study involved interviews with over 2,400 meningioma cases, 2,700 glioma cases and over 5,600 controls in regions of 13 countries, with a primary focus on use of mobile phones. A subset of INTERPHONE study investigators were interested in investigating occupational risk factors and had agreed to include in the interview questionnaire a module to elicit a detailed lifetime job history. That module was used in seven of the INTERPHONE countries, and the subjects in those countries comprise the INTEROCC study subjects. No further data collection was required from the study subjects. The second pillar for this project, the FINJEM, allowed us to infer exposure to many occupational agents. Following examination of the agents that were available in FINJEM, and a review of previous epidemiologic evidence concerning occupations and brain cancer risk, we established a list of 28 agents that would be worthwhile to evaluate in relation to brain cancer. These 28 substances comprised solvents, metals, combustion products, dusts and other chemical agents. For most of them there is little epidemiologic evidence, and little or no literature on possible mechanisms of brain cancer carcinogenesis. This should not be an impediment to studies such as the present one, since most known carcinogens were discovered by epidemiologic or clinical observation, before there were evidence-based biological plausibility arguments [30]. The present article focuses on risks of glioma in relation to 10 of the agents, namely combustion products (diesel exhaust emissions, gasoline exhaust emissions, benzo(a)pyrene), dusts (asbestos, crystalline silica, wood dust, animal dust) and certain other chemical agents (formaldehyde, oil mist, sulphur dioxide). Subsequent papers will address risks related to other agents and to meningioma.

## Methods

### The INTERPHONE study

INTERPHONE was a population-based case–control study of brain cancer carried out in 17 centers in regions of 13 countries [8,28]. The main purpose was to investigate possible associations with use of mobile phones. For most centers, eligible cases were all patients aged between 30 and 59 years and diagnosed with a glioma or meningioma tumor between 2000 and 2004. In Germany and the UK, the upper age limit was 69 years. Israel had no upper age limit for recruitment; however, only subjects aged 30 to 69 years were included from this country in these analyses. Population controls were frequency or individually matched to cases by sex, age (within 5 years) and center. The sampling frame for population controls differed from center to center. Each of the following was used in at least one center: electoral list, population registry, random digit dialing, general practice patient lists and national health insurance plan lists. When possible, a face-to-face interview with the study subjects was conducted by a trained interviewer using a computer-assisted questionnaire. When the study subject had died or was too ill to be interviewed, the interview was conducted with a proxy respondent, usually the spouse, but occasionally an offspring. While this occurred not infrequently among cases, it occurred rarely among controls when the control was selected from the sampling frame, but was not well enough to conduct the interview. Detailed information was collected on socio-demographic characteristics, lifetime use of mobile phones and medical history. Details of each center’s study methods are described elsewhere [8,28].

In addition to the core questionnaire, a subset of INTERPHONE study investigators had agreed to include in the interview questionnaire a module to elicit a detailed lifetime job history, and to participate in an analysis of occupational risk factors.

All participating study centres obtained the appropriate Institutional Review Board authorisations. Written informed consent for participation in the study was obtained from all participants.

### The INTEROCC study

The detailed occupational history module was used in seven of the INTERPHONE countries (Australia, Canada, France, Germany, Israel, New Zealand and the UK), and the subjects in those countries comprise the INTEROCC study subjects.

The lifetime work history requested information for all jobs held by participants for more than 6 months, and included job title, description of tasks, company name, description of activities of the company, and the start and end year for each job. Each job held by subjects was coded according to international occupation and industry classifications: the International Standard Classification of Occupations editions 1968 (ISCO68) [31] and 1988 (ISCO88) [32], and the International Standard Industrial Classification of All Economic Activities, revision 2 (ISIC71) [33]. Common coding guidelines were provided to each center in order to ensure homogeneity in the coding. An inter-rater trial was conducted at the start of coding and results were discussed with each center in a further effort to ensure consistency of coding [34].

### Occupational exposure assessment

Occupational exposure to the agents was assessed using a modified version of the Finnish job exposure matrix FINJEM [29]. Based on the Finnish occupation classification system containing 311 major occupational groups and covering the calendar period from 1945 to 2003 divided into several sub-periods, FINJEM can translate an occupational history into a history of exposure to about 90 chemical, physical, behavioral, microbiological, ergonomic and psychosocial factors, including the 10 agents under investigation in this paper. FINJEM provides two exposure estimates for each combination of occupation, calendar sub-period, and agent: the proportion of workers in that occupation who were considered to be exposed to the agent (P) and the mean level of exposure among the exposed (L) expressed in concentration units. When P was considered to be less than 5%, the level in FINJEM was set to zero. The estimates of P and L were based on exposure measurements, hazard surveys, and the judgements by Finnish occupational hygienists. The fraction of daily or weekly worktime during which the worker is thought to be exposed to the agent is implicitly taken into account because L was constructed as a time-weighted average. All 10 agents considered in this paper are dusts or gases or fumes that entailed respiratory exposure.

Since FINJEM uses the Finnish occupational coding system and the INTEROCC work histories were coded according to international classifications, it was necessary to develop a “crosswalk” between the Finnish codes and the ISCO68. Furthermore, for our purposes, FINJEM was modified in three ways [35]. 

First, the time window 1960–1984 was split into pre and post-1974 periods, and some specific FINJEM entries were modified for some of the agents of interest in order to increase consistency and specificity of exposure assessment. Second, exposure to benzo(a)pyrene was modified to include exposure to environmental tobacco smoke in the workplace. Third, meetings were held with occupational exposure experts from the different centers in order to consider possible differences in the meaning of different job titles across countries and to consider possible occupations and industries where the FINJEM might not be generalizable to other countries. The resulting changes were embodied in a revised version of FINJEM that we call INTEROCC JEM [35].

### Statistical methods

In this article, we are focusing on glioma only. All interviewed controls in the seven INTEROCC countries were used in these analyses, including those recruited for meningioma cases in the centers which used individual matching.

For each agent, three exposure indices were defined for the main analysis: i) ever *vs.* never; ii) lifetime cumulative exposure; and iii) total duration of exposure. As noted above, for each job, the INTEROCC JEM provides the probability (P) that a worker in that occupation was exposed to that agent. There are several approaches for deriving an “ever exposure” variable from the INTEROCC JEM. If we include in the definition all those who had a P greater than 5%, it would be very sensitive but most of the subjects labelled as exposed would have had a low probability of exposure. At the other extreme, if we define a high threshold, say 95%, then it would be very specific, but a large fraction of workers truly exposed would be labelled as unexposed. The trade-off between sensitivity and specificity also has implications for the estimated prevalence of exposure. In order to give greater weight to sensitivity than specificity, but not to exaggerate this choice unduly, we used as the *a priori* threshold P ≥ 25%. Thus, ever exposure to a given agent was defined as having held at least one job with a probability of exposure of at least 25% and for at least one year. Subjects who had held jobs with a probability of exposure of less than 25% but greater than 5%, or for less than one year were considered to be of “uncertain” exposure status and were not included in the analyses. The lifetime cumulative exposure index was defined among ever exposed (corresponding to the ever definition criteria) as the sum of the product of the probability of exposure (P), the level of exposure (L) and the duration for each job held by a subject. The continuous cumulative exposure index was categorized according to tertiles of the distribution among exposed controls. The total duration of exposure was defined as the sum of the duration of exposure for each job held by a subject (corresponding to the ever definition criteria) minus the possible overlap period between two jobs. The total duration of exposure was also categorized, but categories were defined *a priori*: 1–4 years, 5–9 years, and 10 or more years of exposure.

To allow sufficient time between occupational exposure and disease initiation, all exposures that had occurred within five years of the reference date (age at diagnosis for cases and age at interview for controls) were not taken into account, therefore establishing a lag period of 5 years.

For all analyses, the reference category included subjects who had never been exposed to the specific chemical agent of interest, e.g. subjects never exposed or exposed with a probability lower than 5%. Because of the exploratory nature of these analyses, each chemical agent was considered independently of exposure to other agents. That is, the possibility of mutual confounding among these agents was ignored.

Associations between glioma and each of the 10 chemical agents were estimated using conditional logistic regression stratified by sex, age (5-year categories) and center. Further, all analyses were *a priori* adjusted for the following variables: i) age as a continuous variable (in order to remove any residual confounding due to age in the strata definition), ii) the maximum education level attained by the subject or her/his spouse (primary, intermediate college, tertiary), iii) the Standard International Occupational Prestige Scale (SIOPS) [36], iv) history of atopy, defined as ever diagnosed with allergy, asthma and/or eczema (recognized as associated with glioma) [37,38], and v) the respondent status (subject himself *vs.* proxy respondent). Education level and SIOPS provide two different measures of socio-economic status.

Missing values for the maximum level of education attained in the household were imputed to the middle category “intermediate college” (14 subjects). Missing values for the SIOPS variable were imputed to the median value in the corresponding subject strata of age (5-year categories), sex, center and maximum level of education attained in the household (104 subjects).

### Sensitivity analyses

Our definition of “ever exposed” embodied *a priori* decisions about probability of exposure (P ≥ 25%), duration (D ≥ 1 year), and lag period (5 years). To evaluate the sensitivity of results to these decisions, we tried different thresholds. For probability of exposure, we implemented thresholds of 5%, 25% (default) and 50%. For duration, we implemented thresholds of 1 year (default) and 5 years. Finally, for lag period, we implemented thresholds of 1 year, 5 years (default) and 10 years. Altogether there were 18 combinations (3×2×3), including the main default combination.

In addition to the 17 sensitivity analyses embodied in those alternative definitions of ever exposure, additional sensitivity analyses were conducted: i) among males and females separately; ii) restricting cases to the subset of glioblastoma cases, iii) restricting the study sample to those who responded for themselves (i.e. excluding proxies), iv) including two additional *a priori* confounders in each model: smoking status (ever, ex, never smokers) and marital status (married *vs.* others); v) excluding each of the a priori confounders from the default model; vi) excluding subjects with missing values, instead of imputing values; vii) for benzo(a)pyrene (which had a high proportion of subjects in the uncertain category), exposure assessment according to the original version of FINJEM, which excluded benzo(a)pyrene exposure from environmental tobacco smoke [29].

## Results

Of the eligible subjects who completed the interview, 54 cases and 36 controls were removed from the analyses because it was technically impossible to link part of their occupational history to the INTEROCC JEM. An additional set of 9 cases and 6 controls were removed due to a family history of neurofibromatosis and/or tuberous sclerosis. Following these removals, the study sample consisted of 1,800 glioma cases and 5,160 controls. Response rates for cases and controls were 68% and 50%, respectively.

Table 1 describes the main characteristics of subjects included in the analysis. Germany, Israel, UK, and Australia are the main contributor countries in terms of number of glioma cases, accounting for more than 80% of the total. The interviews were conducted with proxy respondents for 14% of the cases and less than 1% of the controls. The distribution of medical history of atopic conditions, smoking, marital status, education level, and mean values for SIOPS were similar among cases and controls.

**Table 1:** Main characteristics of glioma cases and controls in the seven-country INTEROCC study

	**Cases (1,800)**		**Controls (5,160)**	
	**n**	**%**	**n**	**%**
**Country**				
Australia	277	15.4	665	12.9
Canada	169	9.4	649	12.6
France	93	5.2	471	9.1
Germany	366	20.3	1494	29.0
Israel	282	15.7	698	13.5
New Zealand	75	4.2	160	3.1
United Kingdom	538	29.9	1023	19.8
**Sex**				
Males	1116	62.0	2308	44.7
Females	684	38.0	2852	55.3
**Age (years)**				
30-39	328	18.2	791	15.3
40-49	460	25.6	1388	26.9
50-59	678	37.7	2022	39.2
60-69	334	18.6	959	18.6
**Interview type**				
Self respondent	1543	85.7	5137	99.6
Proxy respondent	257	14.3	23	0.4
**Atopy**^**a**^				
Yes	416	23.1	1380	26.7
No	1384	76.9	3780	73.3
**Smoking status**				
Current	508	28.2	1427	27.7
Ex	386	21.4	1212	23.5
Never	906	50.3	2521	48.9
**Marital Status**				
Married	1422	79.0	4035	78.2
Single, divorced or widowed	363	20.2	1115	21.6
Unknown	15	0.8	10	0.2
**Maximum level of education attained**				
Primary, secondary	766	42.6	2253	43.7
Intermediate college	382	21.2	1080	20.9
Tertiary	643	35.7	1822	35.3
Unknown	9	0.5	5	0.1

^a^ Atopy: any medical history of allergy, asthma and/or eczema.

Table 2 shows the main results for each of the 10 agents analysed, using the *a priori* cut-point definition of ever exposure: P ≥ 25%, duration ≥ 1 year; lag period of 5 years. Subjects whose exposure probability was greater than 5% but less than 25% were removed from the analysis (number of removed subjects varied by agent).

**Table 2:** Association between 10 occupational agents and glioma in the seven-country INTEROCC study – five year lag period

	**Formaldehyde**						**Oil mist**					
	**Cases (1,800)**		**Controls (5,160)**		**OR **^**d**^	**95% CI**	**Cases (1,800)**		**Controls (5,160)**		**OR**	**95% CI**
	**n**	**% **^**c**^	**n**	**%**			**n**	**%**	**n**	**%**		
**Non exposed**	1586	88.1	4639	89.9	1.0	-	1657	92.1	4810	93.2	1.0	-
**Ever exposed **^**a**^	96	5.3	283	5.5	0.8	0.6-1.1	63	3.5	151	2.9	0.8	0.6-1.1
**Cumulative exposure**^**b**^												
Lowest tertile	32	1.8	99	1.9	0.9	0.6-1.3	12	0.7	50	1.0	0.5	0.3-1.0
Middle tertile	28	1.6	88	1.7	0.8	0.5-1.4	24	1.3	49	0.9	1.0	0.6-1.7
Highest tertile	34	1.9	94	1.8	0.7	0.5-1.1	23	1.3	50	1.0	0.9	0.5-1.6
**Duration (years)**												
1 - 4	28	1.6	106	2.1	0.7	0.5-1.1	14	0.8	52	1.0	0.5	0.3-0.9
5 - 9	22	1.2	85	1.6	0.6	0.4-1.1	22	1.2	47	0.9	1.1	0.6-1.8
≥ 10	44	2.4	90	1.7	1.1	0.7-1.7	23	1.3	50	1.0	0.9	0.5-1.6
	**Diesel exhaust emissions**						**Gasoline exhaust emissions**					
**Non exposed**	1458	81.0	4446	86.2	1.0	-	1558	86.6	4654	90.2	1.0	-
**Ever exposed **^**a**^	201	11.2	427	8.3	1.0	0.8-1.2	191	10.6	415	8.0	1.0	0.8-1.2
**Cumulative exposure**^**b**^												
Lowest tertile	47	2.6	140	2.7	0.7	0.5-1.0	48	2.7	135	2.6	0.8	0.5-1.1
Middle tertile	73	4.1	139	2.7	1.2	0.9-1.7	68	3.8	135	2.6	1.1	0.8-1.5
Highest tertile	77	4.3	143	2.8	1.1	0.8-1.5	71	3.9	139	2.7	1.0	0.7-1.4
**Duration (years)**												
1 - 4	57	3.2	153	3.0	0.8	0.6-1.2	51	2.8	135	2.6	0.8	0.6-1.2
5 - 9	55	3.1	126	2.4	0.9	0.6-1.2	56	3.1	137	2.7	0.8	0.6-1.2
≥ 10	85	4.7	143	2.8	1.3	1.0-1.8	80	4.4	137	2.7	1.3	0.9-1.8
	**Benzo(a)pyrene**						**Sulphur dioxide**					
**Non exposed**	221	12.3	667	12.9	1.0	-	1775	98.6	5124	99.3	1.0	-
**Ever exposed **^**a**^	367	20.4	1056	20.5	0.8	0.6-1.0	20	1.1	19	0.4	2.0	1.0-3.8
**Cumulative exposure **^**b**^												
Lowest tertile	94	5.2	344	6.7	0.7	0.5-0.9	11	0.6	7	0.1	3.1	1.2-8.5
Middle tertile	118	6.6	344	6.7	0.8	0.6-1.1	3	0.2	6	0.1	1.2	0.3-5.0
Highest tertile	149	8.3	353	6.8	0.9	0.7-1.2	6	0.3	6	0.1	1.4	0.4-4.7
**Duration (years)**												
1 - 4	99	5.5	383	7.4	0.6	0.5-0.9	15	0.8	7	0.1	4.4	1.7-11.2
5 - 9	126	7.0	305	5.9	1.0	0.8-1.4	2	0.1	6	0.1	0.3	0.0-2.2
≥ 10	136	7.6	353	6.8	0.8	0.6-1.1	3	0.2	6	0.1	1.1	0.3-4.6
	**Asbestos**						**Crystalline silica**					
**Non exposed**	1352	75.1	4204	81.5	1.0	-	1579	87.7	4712	91.3	1.0	-
**Ever exposed **^**a**^	292	16.2	621	12.0	0.9	0.8-1.1	200	11.1	413	8.0	1.0	0.8-1.2
**Cumulative exposure**^**b**^												
Lowest tertile	89	4.9	210	4.1	0.8	0.6-1.1	54	3.0	135	2.6	0.8	0.3-1.2
Middle tertile	100	5.6	200	3.9	1.1	0.8-1.4	68	3.8	135	2.6	1.1	0.8-1.5
Highest tertile	103	5.7	211	4.1	1.0	0.7-1.3	74	4.1	138	2.7	1.1	0.8-1.5
**Duration (years)**												
1 - 4	84	4.7	219	4.2	0.9	0.6-1.1	52	2.9	135	2.6	0.9	0.6-1.2
5 - 9	104	5.8	191	3.7	1.0	0.7-1.3	61	3.4	136	2.6	0.9	0.7-1.3
≥ 10	104	5.8	211	4.1	1.0	0.8-1.3	83	4.6	137	2.7	1.2	0.9-1.7
	**Wood dust**						**Animal dust**					
**Non exposed**	1706	94.8	4933	95.6	1.0	-	1733	96.3	5006	97.0	1.0	-
**Ever exposed **^**a**^	88	4.9	204	4.0	1.1	0.8-1.5	66	3.7	147	2.8	0.6	0.6-1.2
**Cumulative exposure**^**b**^												
Lowest tertile	31	1.7	66	1.3	1.1	0.7-1.8	18	1.0	49	0.9	0.7	0.4-1.4
Middle tertile	31	1.7	66	1.3	1.1	0.7-1.8	24	1.3	49	0.9	0.9	0.5-1.5
Highest tertile	26	1.4	68	1.3	1.1	0.6-1.7	23	1.3	49	0.9	0.9	0.5-1.5
**Duration (years)**												
1 - 4	32	1.8	69	1.3	1.1	0.7-1.7	25	1.4	55	1.1	0.8	0.4-1.5
5 - 9	27	1.5	63	1.2	1.1	0.7-1.8	17	0.9	43	0.8	0.9	0.5-1.6
≥ 10	29	1.6	68	1.3	1.1	0.7-1.8	23	1.3	49	0.9	0.8	0.4-1.4

^a^ Probability of exposure in the modified version of FINJEM ≥ 25%; duration ≥ 1 year; lag period of 5 years.

^b^ Calculated among exposed subject as ∑Probability × Level × Duration of each job held by a subject; cut-points at tertiles among exposed controls; expressed in mg/m^3^ (oil mist, diesel and gasoline exhaust emissions, crystalline silica, wood dust and animal dust); ppm (formaldehyde, sulphur dioxide); μg/ m^3^ (benzo(a)pyrene) or in fibers/m^3^ (asbestos).

^c^ Percentages may not add up to 100 due to the exclusion of subjects classified as having ‘uncertain’ exposure.

^d^ Odds ratios stratified by sex, age (5-year categories) and center and adjusted for age as a continuous variable, the maximum education level attained in the household, the standard international occupational prestige scale, atopy and the respondent status.

### Combustion products

Lifetime prevalence of exposure to benzo(a)pyrene, diesel exhaust emissions and gasoline exhaust emissions ranged from 8% to 20%. In all cases, prevalence of exposure was much higher for males than females (data not shown).

The patterns of results were similar for the three combustion products. There was no indication of excess risk when analysing ever/never exposure status, and there were no clear dose–response patterns, although the top category of duration to diesel exhaust emissions did manifest a borderline significant OR of 1.3 (95% CI: 1.0-1.8).

We observed generally similar results in all sensitivity analyses, with slight variations in some (data not shown).

### Dusts

The lifetime prevalence of exposure ranged from 4% for wood dust to 16% for asbestos, with intermediate prevalences for crystalline silica and animal dust. As expected, prevalences were much higher for males than females.

None of these agents manifested any association with gliomas, neither in the ever/never analyses, nor in the dose–response analyses, with either cumulative exposure or duration. All sensitivity analyses gave similar results (data not shown).

### Other chemical agents

Prevalence of exposure ranged from 0.4% for sulphur dioxide to 3.5% for oil mist to 5.5% for formaldehyde. Prevalences were much higher for males than females.

ORs for glioma amongst ever exposed compared to never exposed were around 1.0 for both formaldehyde and oil mist, and there were no indications of higher risk in the higher categories of cumulative exposure or duration. For sulphur dioxide, the least prevalent of the 10 agents analysed (thus providing the most imprecise estimates), the OR of glioma for ever exposure was 2.0 (95%CI: 1.0-3.8), but the excess risk was concentrated in the lowest tertile category of cumulative exposure and the lowest duration category.

All sensitivity analyses gave similar results to those presented in Table 2 (data not shown).

To explore whether the borderline significant result regarding sulphur dioxide was in fact reflecting a clearer excess risk in some subset of workers who incurred their exposures in particular occupations or industries, we reviewed the list of jobs of each subject ever exposed to sulphur dioxide. There was no perceptible difference between cases and controls in the distributions of industries and occupations leading to sulphur dioxide exposure. Most of these were in the iron and steel industry. This exploration did not reveal any noteworthy cluster of cases in any particular occupation or industry. Since a borderline significant OR was also observed for one of the subcategories of diesel exhaust emissions, we also examined the lists of jobs of subjects ever exposed to diesel exhaust emissions. There were no discernible differences in occupational profiles between cases and controls, with motor vehicle drivers or mechanics representing the main occupations with diesel exhaust exposure. Here again we did not detect any noteworthy cluster of cases.

All sensitivity analyses are available from the corresponding author upon request.

## Discussion

We believe INTEROCC is the largest study ever conducted on occupational chemical exposure and risk of glioma. We examined the association between glioma and 10 different chemical agents using three different exposure indices. A large number of sensitivity analyses were conducted, varying the definition of exposure as derived from the INTEROCC JEM, covariates and other analytic tactics and stratifying on the nature of the tumor, sex of subjects, respondent status (self/proxy). Considering all the sensitivity analyses, we analyzed over 200 different models for each chemical agent and thus more than 2,000 models in total. Despite the large sample size, none of the 10 agents demonstrated a pattern of findings that would be persuasive of a causal association with glioma. The handful that were borderline statistically significant could most likely be attributed to multiple testing and random chance.

Although there have been few previous studies on occupational risk factors for glioma, in particular, there have been many previous studies on possible associations between brain cancer and occupational circumstances. However, most of these studies have suffered from some combination of low statistical power due to few study subjects combined with rare exposures, and inadequate assessment of occupational exposures. While there have been no consistently and persuasively reported associations, there are a few leads that deserve attention and that have motivated the present analyses.

There have been some reports of possible excess risks of brain cancer among anatomists, pathologists and embalmers [23-25] and this has led to hypotheses regarding a possible role for formaldehyde. But other studies of formaldehyde-exposed workers have failed to support an etiologic association between formaldehyde and brain cancer [39-42]. Our results also failed to support such an association.

While there have been some reports linking PAH exposure to brain cancer risk [43,44], such an association was not observed in all studies [45]. In our study, we focused specifically on benzo(a)pyrene, and our results do not support a possible role of such exposure in the aetiology of gliomas. Still, exposure to PAHs is ubiquitous, occurring in many different occupations and industries, and often in environments where workers are simultaneously exposed to many other agents. Thus, analysing the specific role of PAHs is challenging.

Whereas some previous results suggested a possible increase of brain cancer risk among subjects exposed to wood dust [15,46-48], two large studies, one a cohort study [49] and the other a large international multi-center case–control study [50], failed to detect any risk. In our study, we did not find any association between glioma and wood dust.

For diesel exhaust emissions and gasoline exhaust emissions, an increased risk of brain cancer has been reported among subjects employed as motor vehicle operators [17,51], but our results did not support such an association. The borderline significant OR for one subset analysis of diesel exhaust emissions is most likely due to random chance since we did not observe any excess risks in relation to cumulative exposure.

An association between asbestos exposure and brain cancer risk has been suggested by some studies [47,52], but other investigations, including a recent large multi-center case–control study of brain tumors in adults, did not find any association between gliomas and asbestos or insulation products in both men and women [50]. These findings are in agreement with our results, with ORs near the null.

There has not been previous research on sulphur dioxide in relation to glioma. While we found a borderline statistically significant OR between sulphur dioxide, the least prevalent of the 10 agents, and glioma, this was due to an excess of cases with very short duration, and it is likely to be explained by random variability.

Finally, previous evidence for an association between occupational exposure to oil products and/or animal products and glioma is sparse and inconclusive [43,50], and our results fail to support any such association.

Participation rates were 68% among glioma cases and 50% among controls. While these rates are not atypical for contemporary population-based case–control studies, they are low enough to raise concerns about a potential participation bias. This issue has been explored in some depth in the parent INTERPHONE study with regard to mobile phone use and brain cancer [53], and it was estimated that differential participation rates could those estimated ORs by 10% to 15%. Such biases, related to the differential cellphone usage of participants *vs.* non-participants, are unlikely to be applicable to the present analyses of occupational exposures because whereas cellphone usage is a prevalent behavioural characteristic that might conceivably be related to the behaviour of being willing and available to participate in a study, it is less likely that being exposed to chemicals at work is similarly correlated with study participation. Even if there was a bias in the same order of magnitude, it would hardly change the overall inferences of the present analysis, as the ORs are far from being statistically elevated.

The only solidly established risk factors for glioma are rare (ionizing radiation, rare medical conditions), probably rarer than the agents that we are examining. Also, these risk factors are probably not strongly correlated with the agents we are examining. These conditions militate against the likelihood that there was confounding of our results by known risk factors. Further, since our observed ORs were mainly around the null value, if confounding had occurred it would have distorted a true causal association to a null one, and this would require quite strong negative correlation between the known risk factor(s) and the agents that we are examining. Since there has been some debate about the possible association between socioeconomic status and brain cancer risk [13,26,45,54,55], we adjusted all results for socioeconomic status using two different markers, one based on the Standard International Occupational Prestige Scale and another one based on the maximum level of education attained in the household. In some sensitivity analyses, we also adjusted for marital status (married *vs*. other) and this additional adjustment did not change results. While it is theoretically possible that our essentially null findings were biased by other unmeasured confounders, this seems unlikely, as such factors would have to be quite strongly negatively associated with the occupational agents we examined, conditional on the socio-economic factors we did adjust for.

The exposure assessment was based on the subjects’ reported job histories, complemented by the data in the INTEROCC JEM. The assessments did not rely on self-reports of exposures, which have been shown to be unreliable [56]. Besides the advantage of providing occupational exposure assessments quickly and with modest cost, especially considering the large number of study subjects that had to be evaluated (approximatively 7,000 subjects), the use of a JEM has the advantage of providing a standardized tool to assign exposure and thereby minimizing the possibility of recall and reporting bias. On the other hand, a JEM allocates the same exposure estimate to each subject in a given occupation in a given era, without taking into account inter-individual variability in performed tasks and job environments. The error involved in applying a JEM is non-differential with respect to case/control status and therefore can lead to some attenuation of true odds ratios [57]. Furthermore, in implementing the INTEROCC JEM, we had to deal with values of probability of exposure ranging from 0% to 100%. It was not self-evident how to use this information in developing an index of exposure for our study. We decided to create an ever/never exposed variable by dichotomising the continuous probability entries at 25% probability of exposure as assessed by the INTEROCC JEM. Such a decision, favouring sensitivity over specificity, is debatable and can lead to further exposure misclassification [58]. In sensitivity analyses, we used 5% and 50% as alternative cut-points, and the results were almost identical. Higher values of the cut-point were impractical since the number of subjects considered exposed would be too low to sustain valid meaningful analyses.

A final source of uncertainty and error relates to the generalizability of the exposure profiles from Finland to other countries. There must surely be some variability between countries in the exposures of workers. Firstly, the industrial profiles differ between different countries. But this in itself is not a source of error in our study. What would cause error is the fact that even within a given occupation, workers in different countries may experience different exposures. All of the countries in INTEROCC have modern industrial economies where, *a priori*, we would not expect industrial processes to be radically different within occupations as would be the case if we were comparing countries at very different levels of industrialisation. Two comparisons have been carried out between FINJEM estimates and those derived by local experts, one in Australia [59] based on a set of exposure agents that were not part of the present analysis, and one in Canada [60], based on a set of exposures that included those analysed here. Both comparisons demonstrated varying degrees of concordance between exposure estimates derived from FINJEM compared with those provided by local experts. For some agents there was high concordance, while for other agents, the concordance was rather low. An indirect indication that our results were not compromised by inter-country patterns of exposure in relation to occupations is the fact that when we carried out analyses within countries, the results were the same as when we combined countries (data not shown).

Statistical power to detect risks was limited by the low prevalence of exposure for some agents, by the measurement error inherent in our exposure assessment, and by the fact that many of the exposed subjects may have experienced relatively low concentrations of exposure. Thus, the essentially null results presented here should not be interpreted as strong evidence for an absence of any risk. Still, this is the strongest evidence to date on glioma risks from these agents.

## Conclusion

This is the largest study to date to investigate associations between selected occupational exposures and glioma. Our findings do not support an association between any of the chemical agents examined and risk of glioma.

## Competing interests

The authors declare that they have no competing interests.

## Authors’ contributions

AL: analysis and manuscript writing. JP: involved in exposure assessment, analysis strategy for the INTEROCC study. LR: coordinator involved in study design, exposure assessment, data management, statistical analysis, and manuscript development. EC: responsible for the coordination of the INTEROCC study, supervision of database management, validations, validation of exposure coding, estimation of exposure for study subjets, participation in analyses and in interpretation of results of INTEROCC, JS: overall supervision of all aspects of this manuscript, including analytical approach, choice of exposure parameters and interpretation of the findings. Other co-authors: responsible for original data collection and/or for the decisions taken about exposure assessment and analysis strategy, as well as review of the manuscript. All authors read and approved the final manuscript.

## Pre-publication history

The pre-publication history for this paper can be accessed here:

http://www.biomedcentral.com/1471-2458/13/340/prepub
